# Thrombospondin-1 might be a therapeutic target to suppress RB cells by regulating the DNA double-strand breaks repair

**DOI:** 10.18632/oncotarget.6835

**Published:** 2016-01-07

**Authors:** Pei Chen, Na Yu, Zhang Zhang, Ping Zhang, Ying Yang, Nandan Wu, Lijun Xu, Jing Zhang, Jian Ge, Keming Yu, Jing Zhuang

**Affiliations:** ^1^ State Key Laboratory of Ophthalmology, Zhongshan Ophthalmic Center, Sun Yat-sen University, P. R. China 510060

**Keywords:** DNA double strand breaks, histone deacetylation, retinoblastoma, Thrombospondin-1

## Abstract

Retinoblastoma (RB) arises from the retina, and its growth usually occurs under the retina and toward the vitreous. Ideal therapy should aim to inhibit the tumor and protect neural cells, increasing the patient's life span and quality of life. Previous studies have demonstrated that Thrombospondin-1 (TSP-1) is associated with neurogenesis, neovascularization and tumorigenesis. However, at present, the bioactivity of TSP-1 in retinoblastoma has not been defined. Herein, we demonstrated that TSP-1 was silenced in RB cell lines and clinical tumor samples. HDAC inhibitor, Trichostatin A (TSA), could notably transcriptionally up-regulate TSP-1 in RB cells, WERI-Rb1 cells and Y79 cells. Moreover, we found human recombinant TSP-1 (hTSP-1) could significantly inhibit the cell viability of RB cells both *in vitro* and *in vivo*. Interestingly, hTSP-1 could significantly induce the expression of γ-H2AX, a well-characterized *in situ* marker of DNA double-strand breaks (DSBs) in RB cells. The DNA NHEJ pathway in WERI-Rb1 cells could be significantly inhibited by hTSP-1. A mutation in Rb1 might be involved in the hTSP-1-medicated γ-H2AX increasing in WERI-Rb1 cells. Furthermore, hTSP-1 could inhibit RB cells while promoting retinal neurocyte survival in the neuronal and retinoblastoma cell co-culture system. As such, TSP-1 may become a therapeutic target for treatment of retinoblastoma.

## INTRODUCTION

Retinoblastoma is a pediatric eye tumor arising in the retina, representing the most common childhood intraocular malignancy. Despite a good understanding of its etiology, mortality from retinoblastoma is approximately 70% in countries of low and middle income. Worldwide, most of the estimated 9000 newly diagnosed patients every year will unfortunately die [1]. Currently, several treatments are available for retinoblastoma, including chemotherapy, radioactive plaque, external beam radiotherapy, cryotherapy and surgery [2]. However, each of these therapies has major drawbacks for pediatric patients. Ideal therapeutic agents for the treatment of tumors should possess the following properties: inhibition of the proliferation of tumor cells and protection of neurons against the excitotoxicity induced by retinoblastoma cells. New adjuvant treatments with better safety and efficacy profiles are needed for retinoblastoma.

TSP-1, a matricellular protein, plays multiple roles in tumor development [3, 4]. It is clear that TSP-1 is involved in inhibiting angiogenesis both *in vitro* and *in vivo* [4–5]. However, the expression level of TSP-1 is different in divergent types of tumors. For example, TSP-1 is highly expressed in the cells of thyroid cancer, breast and colon cancer, and glioma [6–9]. In contrast, TSP-1 is silenced in a subset of undifferentiated, advanced-stage tumors and neuroblastoma cell lines [10]. Currently, the expression level of TSP-1 in retinoblastoma remains unclear, although some studies have indicated that TSP-1 is present in the intraocular fluids and drainage pathway, where it might function in maintaining the anti-angiogenic environment and in intraocular pressure control, respectively [11].

Moreover, the role of TSP-1, which has been identified either as a tumor suppressor or as a tumor promoter, in cancer progression remains controversial [4]. Some studies have demonstrated that TSP-1 promotes tumor growth by enhancing cell migration, invasion and proliferation [12, 13]. TSP-1 promoted tumor cell invasion and metastasis by cooperating with VEGF, FGF2, and TGF-β2 [14, 15]. TSP-1 levels were higher in patients with advanced breast cancer *vs*. patients with early breast cancer [16]. Elevated secretion of TSP-1 has sometimes been considered a predictive factor in diagnosis [3]. However, multiple evidences exist indicating that TSP-1 inhibited angiogenesis by direct effects on endothelial cell migration and survival by activating CD36, caspase-3 and VEGFR-2 [17, 18]. Conflicting results have been obtained, indicating that TSP-1 could paracrinically inhibit tumor angiogenesis and suppress the growth of solid tumors by the BMP4/thrombospondin-1 loop [19]. In addition, TSP-1 might be associated with DNA damage. TSP-1 could stimulate phosphorylation of p47 and increase production of superoxide, which induced cell death [20]. TSP-1 signaling could correspondingly increase high radiosensitivity. Conversely, soft tissues in TSP-1-null mice are remarkably resistant to radiation injury [21]. Guo's study directly demonstrated that intact TSP-1 and its active type I repeat peptides could cause DNA disability and result in notably DNA fragments in endothelial cells [22]. Moreover, one patent related to TSP-1 in cancer therapy has been approved by the U.S. FDA [23]. Knockdown of TSP-1 resulted in a reduction in the adhesion and migration/invasion of human thyroid cancer cells [6]. Currently, the bioactivity of TSP-1 in retinoblastoma has been not well defined.

Furthermore, more evidence has indicated that TSP-1 plays an important role in neural development and neural protection [24]. Blake *et al* reported that TSP-1 promoted neural cell migration by binding to ApoER2 in postnatal neuronal migration [25]. TSP-1 astrocyte-secreted proteins could promote CNS synaptogenesis [26, 27]. TSP-1 is necessary for synaptic plasticity and functional recovery after stroke [28, 29]. Additionally, our previous study proved that TSP-1 secreted by bone marrow stromal cells could contribute to retinal ganglion cell neurite outgrowth and survival [30]. The treatment of retinoblastoma by surgery or other procedures often causes damage to the neurocytes of the retina. Therefore, determining the bioactivity of TSP-1 in retinoblastoma might be helpful not only for tumor therapy but also for retinal protection.

Based on the evidence above, we sought to determine the expression profile and bioactivity of TSP-1 in retinoblastoma cells both *in vitro* and *in vivo* conditions, and examined the possible underlying mechanisms of TSP-1-mediated anti-retinoblastoma action.

## RESULTS

### TSP-1 is silenced in clinical RB tumor samples and RB cells and histone deacetylation might be involved in this process

**Figure 1:**
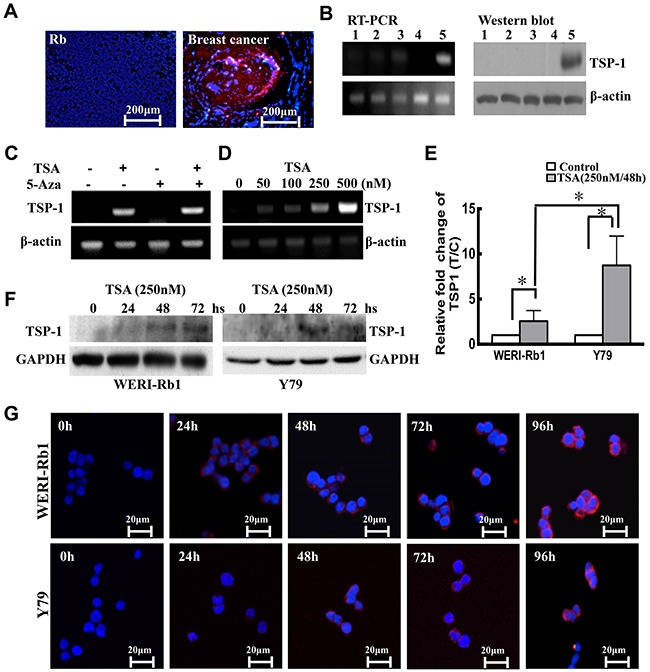
TSP-1 is silenced by histone deacetylation **A.** Immunocytofluorescence showed that compared to the positive control, a lobular type of human breast cancer tissue sample, TSP-1 (red) was lacking in the human retinoblastoma. Original magnification, X 200. **B.** TSP-1 was not detectable in the 3 clinical human RB tumors (lane 1, 2, 3) and WERI-Rb1 cells (lane 4) compared to Hela cells (lane 5) by RT-PCR and Western blot assay. **C.** Only TSA induced expression of TSP-1 in WERI-Rb1 cells. **D.** TSP-1 was induced by TSA in a dose-dependent manner in WERI-Rb1 cells. **E.** TSP-1 levels in WERI-Rb1 and Y79 cells treated with TSA were analysed by real-time PCR. **F.** Western blot analysis of TSP-1 after TSA treatment. GAPDH was shown as an internal control. **G.** WERI-Rb1 and Y79 cells were stained by TSP-1 (red) at different time after treated with TSA (250 nM).

We first measured the expression level of TSP-1 in 14 RB tumor samples diagnosed and verified by oncologists. A lobular type of human breast cancer tissue sample was used as a positive control. Our results showed that TSP-1 was silenced in the human retinoblastoma, whereas it was expressed in the human breast cancer (Figure 1A). Moreover, we measured TSP-1 expression level in other 3 samples and WERI-Rb1 cells by RT-PCR and western blot. As shown in Figure 1B, TSP-1 was absent in the three clinical RB samples (Line1-3) and WERI-Rb1 cells (Line 4), compared to Hela cells (Line 5).

Epigenetic mechanisms have been shown to be responsible for the silencing of TSP-1 in a variety of human cancers [10, 31]. Thus, to examine the role of DNA demethylation and histone deacetylase activity played in the silencing of the TSP-1 gene, WERI-Rb1 cells were treated with the demethylating agent 5-Aza-dC and the histone deacetylase inhibitor TSA, alone or in combination. Our results showed that TSP-1 was notably induced by TSA (500 nM) in WERI-Rb1 cells, whereas treatment with 5-Aza-dC (5 μM) had no effect on TSP-1 expression (Figure 1C). As shown in Figure 1D, TSP-1 was induced by TSA in a dose-dependent manner. WERI-Rb1 cells treated with 250 nM TSA markedly expressed TSP-1, compared to the controls by RT-PCR assay. To further validate the expression level of TSP-1 in the retinoblastoma cells, we analyzed its expression level in two kinds of RB cell lines, WERI-Rb1 and Y79. Real-time RT-PCR assay indicated the mRNA level of TSP-1 was barely detectable in both WERI-Rb1 and Y79 cells, however, significantly up-regulated upon 250 nM TSA treatment for 48 hours (Figure 1E). Moreover, TSA has shown stronger effect in inducing the expression of TSP-1 on Y79 cells (by 3.25-fold) than on WERI-Rb1 cells (by 1.18-fold) (*p<0.05). Furthermore, western blot and immunohistofluorescence assay also demonstrated that the expression level of TSP-1 in both WERI-Rb1 and Y79 cells was time-dependently increased after treatment with TSA (Figure 1F and 1G).

**Figure 2:**
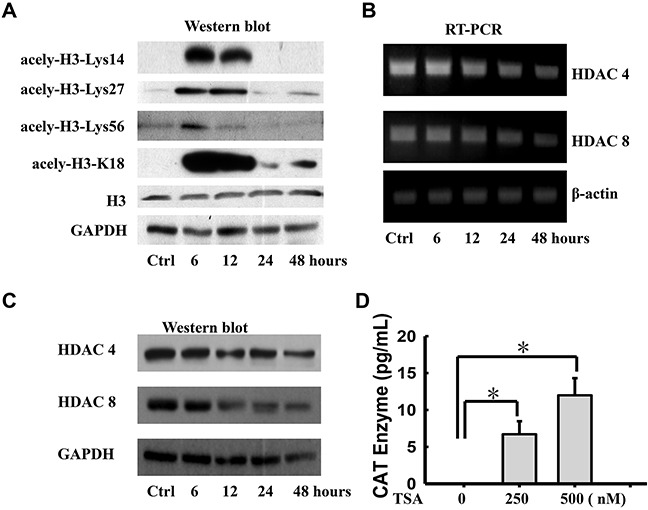
Histone deacetylase inhibitor transcriptionally induces expression of TSP-1 in WERI-Rb1 cells **A.** Acetylated histone H3 (Lys14, 27, 56 and K18) protein levels were increased in TSA-treated (250 nM) WERI-Rb1 cells compare to the total histone H3. Analysis of RT-PCR **B.** and Western blot **C.** showed that TSA (250 nM) notably induced the expression of HDAC4 and HDAC8 in WERI-Rb1 cells. **D.** CAT enzyme elisa of WERI-Rb1 cells treated with TSA for 24 h when transfected with the plasmid containing −2033/+750 human TSP-1 promoter. β-actin or GAPDH was shown as an internal control. All results were confirmed in three independent experiments. *Statistically significant differences between the TSA and control (*p<0.05).

Moreover, we examined acetylated histone H3, as well as total histone H3 levels, to demonstrate that the inhibition of histone deacetylase by TSA resulted in a global increase in histone acetylation. Western blot was performed to assay the expression of acetylated histone H3, acetylated at Lys14, 27, 56 and K18, in WERI-Rb1 cells after treatment with 250 nM TSA at different time points. As shown in Figure 2A, the basal levels of acetylated histone were quite low in the control cells, compared to the expression of histone H3. Acetylated histone H3, at Lys14, 27, 56 and K18, was dramatically increased at 6 h and 12 h and then decreased to basal levels at 24 h in TSA-treated WERI-Rb1 cells, compared to the untreated cells. To determine which HDAC isoform was responsible for the induction of TSP-1 gene expression, we also examined the effects of treatment with the HDAC inhibitor TSA on the expression of the various HDAC genes by RT-PCR assay. As shown in Figure 2B, TSA remarkably reduced the expression levels of HDAC4 and HDAC8. Except for the expression changes in HDAC4 and HDAC8, the expression of the other HDAC genes was not altered to a significant extent (data not shown). Western blot assay also confirmed this outcome (Figure 2C).

To further define that histone deacetylation is involved in TSP-1 silencing, WERI-Rb1 cells transfected with the construct, which contains human TSP-1 promoter sequences from −2033 to +750 and a chloramphenicol acetyltransferase (CAT)-encoding reporter gene, were treated with 250 or 500 nM TSA and were processed for CAT assay. As shown in Figure 2D, TSA significantly increased CAT activity in WERI-Rb1 cells in a dose-dependent manner, compared to the controls (Control, 0; 250 nM, 6.711±1.757; 500 nM, 11.987±4.49; respectively. *p<0.05). Thus, these findings strongly supported that histone deacetylation was responsible for the silencing of TSP-1 in retinoblastoma.

### hTSP-1 inhibits cell viability and induces cell cycle arrest in WERI-Rb1 cells *in vitro*

**Figure 3:**
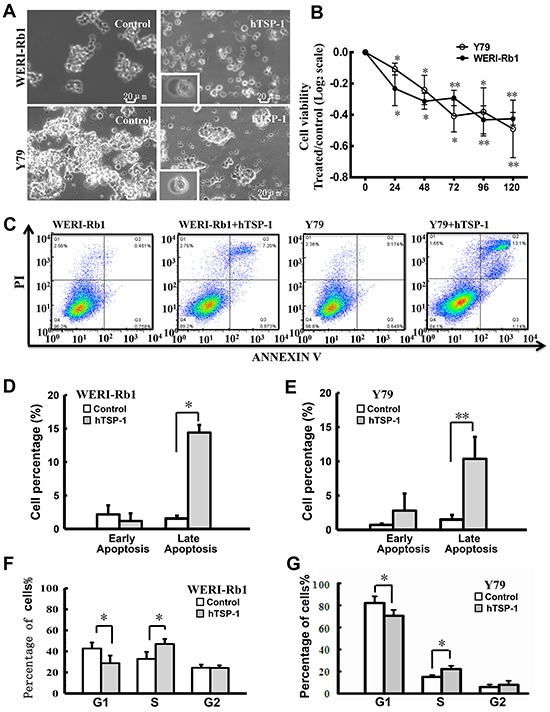
hTSP-1 affects cell viability and the cell cycle of WERI-Rb1 and Y79 cells **A.** The morphology changes of WERI-Rb1 and Y79 cells treated with hTSP-1 (25 nM) for 120h. The cells showed membrane blebs (bottom left pictures) after hTSP-1 treatment. **B.** hTSP-1 inhibited the viability of WERI-Rb1 and Y79 cells compared to negative controls. The results of the CCK8 assays were plotted on a log2 scale. **C.** The early apoptosis and cell death of WERI-Rb1 and Y79 cells was quantified by flow cytometry after Annexin V and propidium iodide staining. **D, E.** The percentages of early and late apoptotic WERI-Rb1 and Y79 cells are presented in histograms. The data showed that hTSP-1 had no effect on the incidence of early apoptosis, however, could markedly increase the incidence of late apoptosis in both of the cells. **F, G.** hTSP-1 impaired S-phase progression in both WERI-Rb1 cells and Y79 cells after hTSP-1 treatment for 24 h compared to the control. In addition, there was no significant difference in the G2 phase G2/M in WERI-Rb1 cells and Y79 cells. *Statistically significant differences between the TSA and control (*p<0.05, **p<0.01).

TSA could induce the re-expression of TSP-1 in both WERI-Rb1 cells and Y79 cells. However, TSA induced global acetylation in cells, which affected a massive number of genes. Thus, to evaluate the exact effects of TSP-1 on retinoblastoma cells, in parallel, hTSP-1 was added to the medium of the WERI-Rb1 cells and Y79 cells. Figure 3A shows the microscopic photographs of WERI-Rb1 cells and Y79 cells treated with 25 nM hTSP-1 for 120 h. Both the WERI-Rb1 and Y79 cells treated with hTSP-1 were much more scattered than the controls, which characteristically grew in loose, grape-like clusters. Furthermore, many cells presented massive membrane blebs among the cells treated with hTSP-1, while such blebs were completely absent in the untreated control cells, which might be a characteristic feature of apoptosis as part of a necrotic process. The growth-inhibitory effects of hTSP-1 on the human retinoblastoma cell lines WERI-Rb1 and Y79 was evaluated *in vitro* at different time points using a CCK8 assay. Cell viability was represented by quantile normalization in log2 scale. As shown in Figure 3B, the viability of WERI-Rb1 cells treated with hTSP-1 was significantly reduced continuously (hTSP-1, 24 h, −0.232±0.109; 48 h, −0.313±0.050; 72 h, −0.292±0.049; 96, −0.432±0.089; 120 h, −0.425±0.041, respectively), compared to the controls (Control, 0) (*p<0.05, **p<0.01), demonstrating that hTSP-1 could significantly inhibit the growth of WERI-Rb1 cells. Consistently, incubation with the exogenous hTSP-1 could also markedly inhibit the cell viability of Y79 cells (hTSP-1, 24 h, −0.107±0.038; 48 h, −0.241±0.094; 72 h, −0.408±0.102; 96, −0.381±0.114; 120 h, −0.489±0.083, respectively) (*p<0.05, **p<0.01).

In addition, the incidence of early and late apoptosis induced by hTSP-1 in both WERI-Rb1 cells and Y79 cells was quantified by flow cytometry after Annexin V and PI staining. As shown in Figure 3C and 3E, TSP-1 could induce apoptosis in RB cells. Late but not early apoptosis was significantly induced in both WERI-Rb1 cells and Y79 cells followed 48 hours incubation with hTSP-1 (For WERI-Rb1, Early apoptosis: hTSP-1, 1.496±0.69%, Control, 0.72±1.96%, p>0.1; Late apoptosis: hTSP-1, 10.366±3.2%, Control, 2.8±2.5%, *p<0.05) (For Y79, Early apoptosis: hTSP-1, 1.547±0.43%, Control, 2.166±1.36%, p>0.1; Late apoptosis: hTSP-1, 14.4±1.14%, Control, 1.176±1.15%, **p<0.01). Moreover, as shown in Figure 3F, there was a decrease in the number of WERI-Rb1 cells in the G0/G1 phase (hTSP-1, 28.74±7.30%, Control, 42.74±5.70%, *p<0.05) and an increase in the number of cells in the S phase (hTSP-1, 47.00±4.83%, Control, 32.83±6.63% *p<0.05), whereas there was no significant difference in the number of cells in the G2 phase (hTSP-1, 24.26±2.48%, Control, 24.43±2.97%, P>0.1). Similar result was obtained from Y79 cells treated with hTSP-1, demonstrating an S phase arrested (G0/G1 phase: hTSP-1, 68.56±2.42%, Control, 82.81±6.11%, *p<0.05. S phase: hTSP-1, 22.19±3.00%, Control, 15.11±1.68%, *p<0.05. G2 phase: hTSP-1, 8.47±2.92%, Control, 5.88±2.33%, p>0.1) (Figure 3G). These results showed that hTSP-1 could induce the impairment of cell cycle progression and induce S-phase arrest in WERI-Rb1 cells and Y79 cells. Taken together, these results indicated that TSP-1 could also significantly affect proliferation and viability and thus might be a potential therapeutic candidate for the treatment of resistant retinoblastoma.

### hTSP-1 might affect the repair of DNA DSBs by attenuating the NHEJ of WERI-Rb1 cells

**Figure 4:**
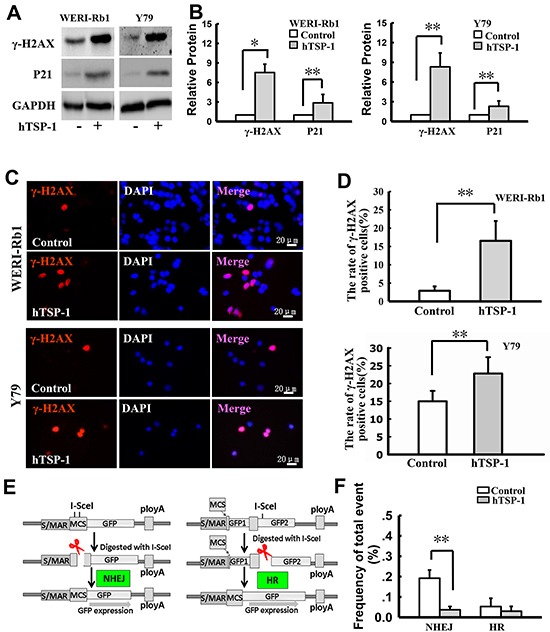
hTSP-1 induces DNA double-strand breaks and affects the NHEJ in WERI-Rb1 and Y79 cells **A.** Western blot analysis of γ-H2AX and P21 in WERI-Rb1 and Y79 cells with or without hTSP-1 treatment for 48 h. GAPDH was shown as an internal control. **B.** Relative quantification of expression of γ-H2AX in WERI-Rb1 and Y79 cells was quantified by densitometry. **C.** γ-H2AX (red) was present on WERI-Rb1 and Y79 cells treated with hTSP-1. **D.** The relative quantification of γ-H2AX expression following hTSP-1 was determined by counting foci in 50 randomly positive cells, and these data were graphically represented, and indicated that hTSP-1 significantly increases DNA DSBs in WERI-Rb1 and Y79 cells. **E.** The structure of the NHEJ and HR substrate and the strategy to measure NHEJ and HR. **F.** Overall I-SceI-induced DSB end-rejoining efficiency in WERI-Rb1/pEPI-NHEJ and WERI-Rb1/pEPI-HR with or without hTSP-1 in WERI-Rb1, as measured by flow cytometry. Significant difference in the repair efficiency of NHEJ with hTSP-1 versus the control treatment was represented as a histogram. Asterisks indicate statistically significant differences between the control and test cells (*p<0.05, **p<0.01).

Previous studies have reported that failure of DNA double-strand breaks (DSBs) repair could induce S-phase arrest in the cell cycle [32]. To elucidate the mechanism of TSP-1-mediated S-phase arrest in RB cells, we analyzed the state of DNA DSBs in cells following exposure to hTSP-1. As shown in Figure 4A and 4B, hTSP-1 could significantly increase the expression of γ-H2AX in both WERI-Rb1 cells and Y79 cell by 7.53-fold and 10.1-fold, respectively, compared to the controls at 48 h after treatment (*p<0.05). In addition, P21, which accumulated at DNA damage sites and was colocalized with γ-H2AX [33], was also up-regulated in WERI-Rb1 cells and Y79 cells treated with hTSP-1 by 2.86-fold and 2.49-fold, respectively, compared to the controls (**p<0.01), consistent with previous report that intact TSP-1 significantly induced increasing of P21 and DNA fragmentation in endothelial cells [20, 34]. Therefore, these results indicated that TSP-1 might be involved in DNA DSBs repair in both WERI-Rb1 cells and Y79 cells. Dual immunofluorescence of γ-H2AX and DAPI confirmed that γ-H2AX was present in most WERI-Rb1 cells as well as Y79 cells treated with hTSP-1 (Figure 4C). The ratio of γ-H2AX foci-positive cells is represented as a histogram. As shown in Figure 4D, hTSP-1 significantly resulted in a marked increase in γ-H2AX staining (WERI-Rb1: 16.53±5.41%, Y79: 22.79±4.62%), compared to the controls (WERI-Rb1: 2.90±1.20%, Y79: 14.98±2.93%, **p<0.01).

DNA DSBs repair includes homologous recombination (HR) and DNA non-homologous end joining (NHEJ) [35]. To reveal whether TSP-1 was involved in both pathways, we further assessed NHEJ and HR activities of WERI-Rb1 cells. The structure of the NHEJ and HR substrate and the strategy to measure NHEJ and HR have been described previously [36, 37] and are depicted in Figure 4E; this strategy was previously developed and has been successfully used to define the roles of BRCA1, Rb1, Mre11, XRCC4 and Ku80 in the control of DNA DSBs repair in human cells [32, 35, 38–40]. For NHEJ, the plasmid pEPI-NHEJ contains a human S/MAR [41], which stably and independently replicates in WERI-Rb1 cells. Moreover, there are two I-SceI recognition sites before the reporter gene GFP. An artificial ATG (ATGART) between the two sites induces a translational shift, hence preventing GFP luciferase reporter gene expression. After digestion with I-SceI, fully complementary cohesive 3-OH single-stranded ends of four bases are produced upon double cleavage. If rejoining of the double-stranded ends by NHEJ occurs, then the intact GFP can be translated and expressed in cells. Alternatively, error-prone NHEJ at either of the single I-SceI sites can disrupt ATGART, which also allows for the expression of GFP. In contrast, precise re-ligation at a single I-SceI site cannot be detected directly by flow cytometry analysis using this system. The prevalence of GFP-positive cells represents the overall proficiency of NHEJ. For HR, the plasmid pEPI-HR is similar to the NHEJ substrate, which contains two GFP cDNA fragments after the promoter: GFP1 occurs at +1 to +400 bp, and GFP2 covers the whole cDNA. There is an inserter between GFP1 and GFP2, which results in the abnormal expression of GFP. After digestion with I-SceI, HR ends are produced. If HR occurs, GFP is expressed by sharing an extensive sequence homology. The prevalence of GFP-positive cells represents the overall proficiency of HR.

To define the role of TSP-1 in the DSB repair pathway, WERI-Rb1 cells carrying the episomally replicating pEPI-NHEJ or pEPI-HR substrate were treated with 25 nM hTSP-1. At twenty-four hours after treatment, the cells were transfected with the I-SceI endonuclease-expressing plasmid. At 48 h after transfection, the cells were harvested and analyzed by flow cytometry. Comparison with the data in Figure 4F demonstrated that the relative efficiency of NHEJ in the WERI-Rb1 cells treated with hTSP-1 (0.192±0.040%) was significantly lower than in the controls (0.037±0.016%) (*p<0.05). In contrast, the rejoining levels in the cells revealed that hTSP-1 had no differential effects on HR in WERI-Rb1 cells (hTSP-1, 0.053±0.040%; control, 0.030±0.024%, p>0.05). Thus, these findings provided direct evidence that TSP-1 might inhibit DNA NHEJ pathway in WERI-Rb1 cells. In addition, we performed NHEJ and HR assay in Y79. However, transfection efficiency in Y97 cells (1%) is notably low than that of WERI-Rb1 cells (12%). The efficiency of DNA DSBs repair in the assay is very low too. Thus, the results from Y79 cells are not significant in NHEJ and HR assay.

### The mutation of Rb1 might be involved in TSP-1-mediated DNA DSBs repair in WERI-Rb1 cells

**Figure 5:**
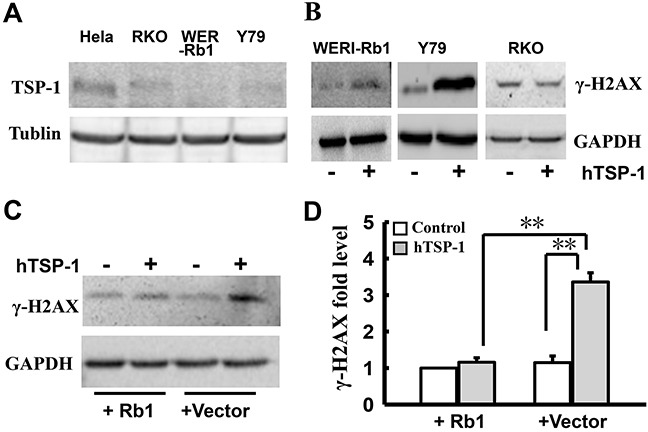
Attenuating DNA NHEJ repair by TSP-1 is specific to WERI-Rb1 cells **A.** Western blot assay showed that TSP-1 was silenced in RKO cells, WERI-Rb1cells and Y79 cells, compared to the positive cells Hela cells. **B.** Western blot assay showed that hTSP-1 did not affect the expression level of γ-H2AX in RKO cells compared with WERI-Rb1 cells and Y79 cells. **C.** Exogenous Rb1 remarkably attenuated hTSP-1-induced increase of γ-H2AX in WERI-Rb1 cells after transfected with pEPI-GFP-Rb1. **D.** Relative quantification of expression of γ-H2AX in WERI-Rb1 cells was quantified by densitometry. The expression of γ-H2AX in WERI-Rb1 cells expressing exogenous Rb1 was not affected compared to the control cells transfected with vector. Exogenous Rb1 significantly attenuated the hTSP-1-induced up-regulation of γ-H2AX in WERI-Rb1 cells. GAPDH was shown as an internal control. Asterisks indicate statistically significant differences between the control and test cells (**p<0.01).

As previous study, the bioactivity of TSP-1-mediated DNA repair is associated with cell type [22]. Therefore, we further investigated whether the mutation of Rb1 is involved in TSP-1-mediated DNA repair in RB cells. We measured γ-H2AX in RKO cells, a line of poorly differentiated colon carcinoma cells. Our data is consistent with the previous report that TSP-1 is silenced in RKO cells by high DNA methylation [42] (Figure 5A). Interestingly, as shown in Figure 5B, hTSP-1 could not alter the expression level of γ-H2AX in RKO cells, compared to that of the controls. Thus, attenuating DNA DSBs repair by TSP-1 might be specific to RB cells.

Because the up-regulation of γ-H2AX TSP-1-mediated occurs in RB cells instead of RKO cells, we speculated that Rb1, a transcription co-regulator, might be involved in this process. To elucidate this hypothesis, WERI-Rb1 cells were transfected with the pEPI-GFP-Rb1 plasmid, expressing wide-type Rb1 and GFP, or the pEPI-vector. GFP-positive cells represented cells expressing exogenous Rb1. Following treatment with hTSP-1, whole lysates of WERI-Rb1 cells with and without exogenous Rb1 were extracted at 48 h post-treatment. Western blot analysis indicated that exogenous Rb1 could significantly attenuate the hTSP-1-induced up-regulation of γ-H2AX in WERI-Rb1 cells (wild-type Rb1, 1.15±0.18), compared to the control cells (vector, 3.36±0.25) (Figure 5C, 5D) (**p < 0.01). The expression of γ-H2AX in Y79 was not changed, which might be caused by low transfect efficiency (data not shown). Therefore, these results suggested that a mutation of Rb1 might be involved in the TSP-1-mediated DNA DSBs repair in WERI-Rb1 cells.

### hTSP-1 could inhibit WERI-Rb1 cells and promote retina neurocyte survival in the neuronal and retinoblastoma cell co-culture system

**Figure 6:**
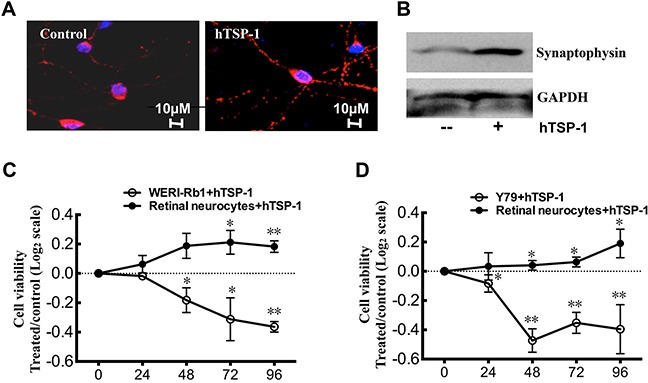
hTSP-1 inhibits WERI-Rb1 and Y79 cells, whereas, promote retina neurocytes survival in co-culture **A.** Immunohistochemical analysis of retina neurons for synaptophysin (red) showed more synaptic puncta, compared to the control. **B.** hTSP-1 increased the expressing level of synaptophysin in retinal neurocytes by western blot assay. **C.** CCK8 assay indicated that hTSP-1 significantly increased the cell viability of retina neurons days compared to the control. Growth of WERI-Rb1 cells was significantly inhibited by hTSP-1 compared to the control. The results of the CCK8 assays were plotted on a log2 scale. **D.** CCK8 assays of retina neurons and Y79 cells co-culture system treated with hTSP-1. The data was plotted on a log2 scale.

Based on previous studies, in which TSP-1 could promote retinal neural survival [28–30], we further defined whether TSP-1 could play the role of a double-edged sword in the inhibition of retinoblastoma growth and the protection of neurocytes. Therefore, a co-culture system was established to mimic physiological conditions, in which WERI-Rb1 cells were cultured in the top chamber and retinal neurons in the bottom chamber. The cells were treated with 25 nM hTSP-1 for 4 days. As shown in Figure 6A and 6B, the expression of synaptophysin, a downstream protein of TSP-1 involved in synaptogenesis [26], in retinal neurocytes was notably up-regulated by TSP-1 according to immunohistochemical analysis and western blot assay (Figure 6A, 6B). Moreover, the results of the CCK-8 assay revealed that the cell viability of neurocytes treated with hTSP-1 was not affected on the first day, compared to the controls. However, at days 2, 3 and 4 after treatment, hTSP-1 could significantly increase the viability of neurocytes, compared to the controls by quantile normalization in log2 scale (hTSP-1, D2, 0.188±0.104, D3, 0.212±0.099, D4, 0.183±0.049, respectively; Control, 0; *p<0.05, **p<0.01)(Figure 6C). In contrast, the growth of WERI-Rb1 cells in the top chamber was significantly inhibited by hTSP-1 beginning on the second day after treatment, compared with the controls (hTSP-1, D1, −0.0178±0.03, D2, −0.182±0.103, D3, −0.312±0.023 and D4, −0.364±0.044, *p<0.05, **p<0.01)(Figure 6C). Moreover, hTSP-1 exerted significantly neuroprotective activity and inhibition effect on Y79 cells too (For retinal neurons: hTSP-1, D1, 0.034±0.093 D2, 0.042±0.033; D3, 0.064±0.034; D4, 0.190±0.098, respectively; Control, 0; *p<0.05; For Y79: D1, −0.083±0.059, D2, −0.473±0.079; D3, −0.352±0.072; D4, −0.415±0.195, respectively; Control, 0; *p<0.05, **p<0.01) (Figure 6D). Therefore, our data indicated that TSP-1 suppressed RB cells growth and had neuroprotective ability in this co-culture system.

### hTSP-1 could inhibit retinoblastoma formation *in vivo*

**Figure 7:**
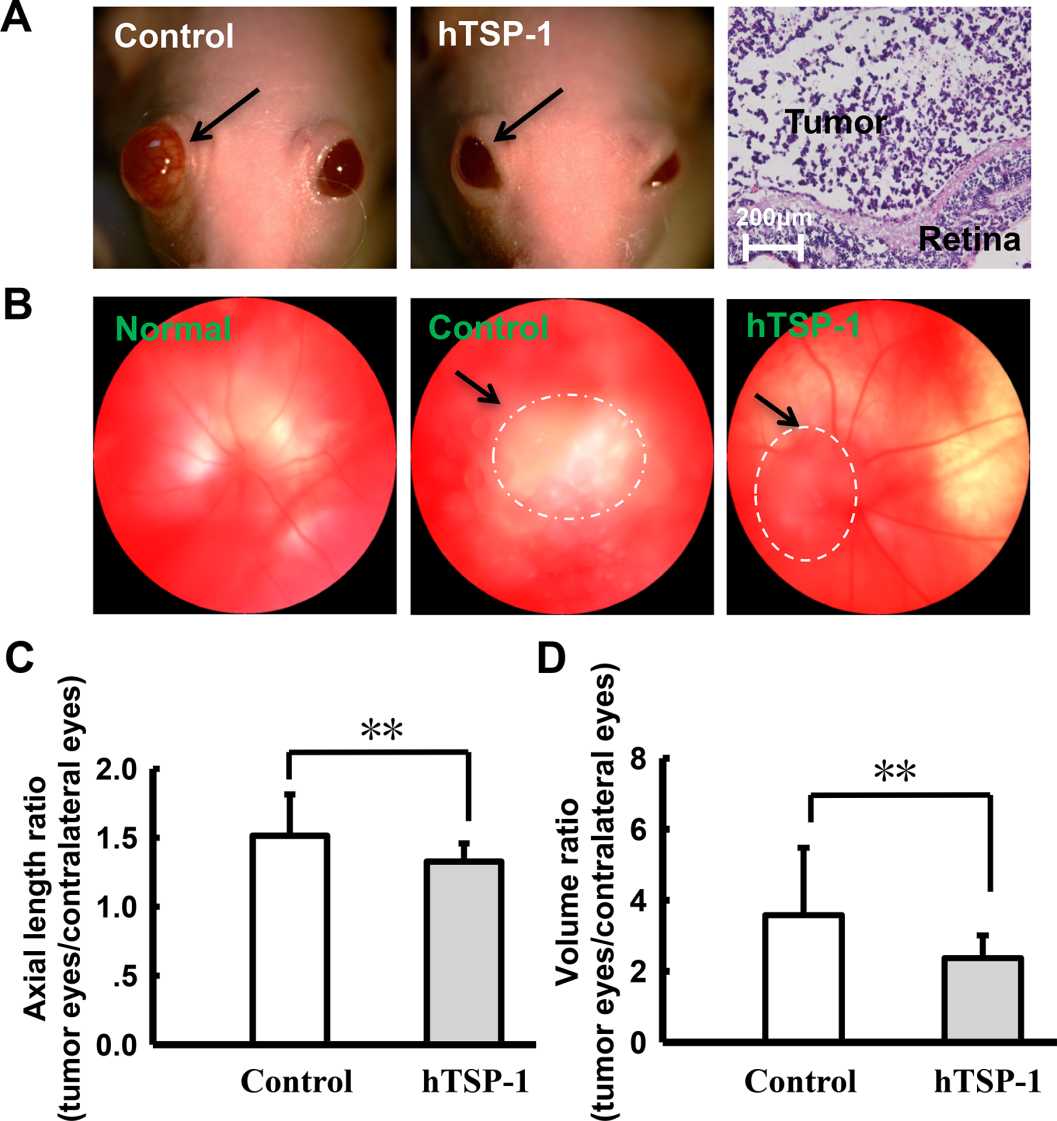
hTSP-1 suppresses the growth of retinoblastoma **A.** photographs of nu/nu nude mice retinoblastoma xenografts. The degree of exophthalmos was significantly decreased after treated with hTSP-1, compared to the control (arrow). **B.** Fundus photograph taken photos of normal, control and hTSP-1 treatment group in 30 days. The images showed that hTSP-1 inhibited tumor growth compared to the control. The axial length **C.** and average eyeball volume **D.** ratio in the control and hTSP-1-treated retinoblastoma at 30 days after implantation of retinoblastoma cells. Asterisks indicate statistically significant differences between the control and test cells (*p < 0.05).

To validate our findings of TSP-1-mediated inhibition of retinoblastoma cells, we performed a tumor-response experiment using our mice xenograft model. A total of 2×10^5^ WERI-Rb1 cells were injected into the vitreous of right eyes. Two weeks after tumor implantation, the nude mice were randomized to different groups receiving injections of hTSP-1 or balanced salt solution for two weeks. Figure 7A showed that the degree of exophthalmos in the WERI-Rb1 implantation mice treated with hTSP-1 was clearly smaller than that of the controls. In addition, hematoxylin and eosin staining of representative retinoblastoma tumor showed the structure of right eye was destroyed. Briefly, fundus photography showed that tumor growth was significantly inhibited in mice treated with hTSP-1, compared with that in the control mice (Figure 7B) (black arrowhead). Because the degree of exophthalmos was positively related to the growth of tumors, we used axial length and volume of the eyeball, which were previously used in clinical and experimental assays [43, 44], to represent tumor growth. In hTSP-1 group, the axial length ratio of eyeballs (L_TSP-1-right_/L_TSP-1-left_) was 1.33±0.132, compared to the controls (L_CON-right_/L_CON-left_), in which it was 1.51±0.301 (**p<0.01) (Figure 7C). Furthermore, the volume ratio of eyeballs (V_TSP-1-right_/V_TSP-1-left_) was 2.37±0.645, and for the control groups, the volume ratio of eyeballs (V_CON-right_/V_CON-left_) was 3.58±1.903 (**p<0.01) (Figure 7D). These data were consistent with our results from WERI-Rb1 cells *in vitro*, suggesting that TSP-1 suppressed tumor growth, compared to the controls.

## DISCUSSION

In the present study, our results showed that TSP-1 might be a therapeutic target to suppress retinoblastoma growth. First, we found that TSP-1 was silenced in RB tumors cells by histone deacetylation. HDAC inhibitor TSA could significantly induce the re-expression of TSP-1 in WERI-Rb1 cells and Y79 cells. hTSP-1 could significantly suppress the growth of RB cells both *in vitro* and *in vivo*. In addition, using a retinoblastoma-neuronal co-culturing system, we also demonstrated that TSP-1 could significantly inhibit RB cells while promoting retinal neurocyte survival. These results indicated therapeutic potential of TSP-1 in the treatment of retinoblastoma. Moreover, we revealed a mechanism, which we believe to be novel, by which TSP-1 could significantly induce γ-H2AX foci, which is a well-characterized in situ marker of DNA DSBs in both WERI-Rb1 cells and Y79 cells. Consistent with role of γ-H2AX, TSP-1 inhibited the DNA NHEJ pathway in WERI-Rb1 cells. Apoptosis assay also demonstrated that TSP-1 could induce apoptosis in RB cells. Furthermore, our study revealed that the mutation of Rb1 might be involved in the TSP-1-medicated DNA DSBs repair in WERI-Rb1 cells. Thus, our present findings provided direct biochemical and functional evidence supporting the notion that TSP-1 might be a therapeutic target to suppress retinoblastoma growth by regulation of DNA DSBs repair.

The first thrombospondin to be identified, TSP-1, is a potent inhibitor of angiogenesis and has complex effects on tumor development [3]. Our data showed that TSP-1 was silenced in both WERI-Rb1 cells and Y79 cells. Moreover, our results indicated that the mechanism of TSP-1 silencing of WERI-Rb1 cells was involved in histone deacetylation, different from that of other neuroblast tumors. Yang *et al* reported that DNA methylation played a dominant role in neuroblastoma cells [10].

TSP-1 appears to play numerous and complex roles in cancer growth and metastasis. The multimodular structure of TSP-1 confers to these molecules anti-tumoral as well as pro-tumoral properties [3, 45]. This apparent discrepancy depends on the cell type or tissue studied. At present, only several studies have demonstrated that TSP-1 is involved in DNA damage. Guo *et al* reported that TSP-1 analogues induced programmed cell death in endothelial cells, based on morphological changes, assessment of DNA fragmentation, and internucleosomal DNA cleavage. Intact TSP-1 also induced DNA fragmentation in endothelial cells [22]. However, the mechanism of DNA repair TSP-1-medicated remains obscure. There is a report indicating that TSP-1 might stimulate reactive oxygen species production by signal-regulatory protein-α in ischemia reperfusion injury. Our data demonstrated that TSP-1 could significantly increase the expression levels of γ-H2AX and P21, which are markers of DNA DSBs in WERI-Rb1 cells and Y79 cells. Moreover, TSP-1 might significantly inhibit DNA NHEJ pathway, whereas it did not affect HR events in WERI-Rb1 cells. Therefore, TSP-1 may be involved in DNA DSBs repair in RB cells.

Interestingly, TSP-1 did not affect the expression of γ-H2AX in RKO cells, indicating that the attenuation of the DNA DSBs repair pathway by TSP-1 was specific to RB cells. Consistent with our hypotheses, exogenous Rb1 could attenuate the increase of γ-H2AX in WERI-Rb1 cells after TSP-1 treatment. Thus, these results indicated that a mutation of Rb1 might be involved in TSP-1-mediated suppression of DNA DSBs repair. Currently, it has been clear that the complex of the Rb1 binding transcript factor E2F plays a crucial role in regulating transcription. Much evidence has demonstrated that the complex of Rb1 and E2F regulates many downstream target genes that are involved in cell cycle progression and DNA replication [46]. However, the direct evidence of Rb1 and TSP-1 in DNA DSBs repair has been not reported yet. One study indicated that the suppression of TSP-1 could be achieved by an alternative mechanism involving the inactivation of both p53 and pRb [47]. In addition, Thrombospondin-1 signaling through CD47 inhibits self-renewal by negative regulating c-Myc and other stem cell transcription factors, such as, c-Myc, Klf4, Oct4, and Sox2 [48]. Therefore, we speculate that TSP-1 is not only a matricellular protein associated with cell migration and invasive, but also a possible transcription factor in regulation of other genes. There might be a complicated mechanism of TSP-1-mediated DNA DSBs repair in retinoblastoma cells, which is required to be investigated in the future.

Furthermore, it is well known that retinoblastoma develops in the retina, which develops rapidly in early life, and results in neural damage [42]. Some reports have indicated that TSP-1 plays a key role in neural development and promoting synaptic activity [24, 49–50]. Synaptic activity could suppress Puma-induced neuronal apoptosis [28]. An increase in synaptic activity could enhance neuronal survival [28, 29]. Compared with WT retina, TSP-1 null retina fails to recover from the laser-induced injury, resulting in irreversible damage [51]. Moreover, TSP-1 counteracts the increased neuronal excitability and neuronal death induced by TNFα. The role of TSP-1 in controlling the balance between excitation and inhibition could facilitate the recovery of normal synaptic activity after injury [52]. Remotely activated ACs promoted the recovery of excitatory input on surviving motor neurons by the up-regulation of TSP-1 expression [53]. Here, using two kinds of co-culture system, our data showed that TSP-1 prolonged retinal neurocyte survival and effectively inhibited the proliferation of WERI-Rb1 cells and Y79 cells. Therefore, TSP-1 might be a potent therapeutic target for suppressing the growth of tumors while protecting normal retina cells.

In summary, this study provided direct biochemical and genetic evidence supporting the notion that TSP-1 is silenced by histone demethylation in retinoblastoma cells. Moreover, TSP-1 could significantly inhibit cell growth of RB cells. Moreover, the expression level of γ-H2AX and P21, well-characterized in situ markers of DNA DSBs, was significantly decreased in RB cells. DNA NHEJ pathway in WERI-Rb1 cells is significantly inhibited by TSP-1 too. Therefore, our results revealed that TSP-1 is not only a potential target gene in the treatment of retinoblastoma but also a mechanism of TSP-1-mediated anti-retinoblastoma effects. Thus, these findings warrant future clinical investigations of the suppression of retinoblastoma and neuroprotection with TSP-1 during retinal irradiation in the pediatric population.

## MATERIALS AND METHODS

### Ethics statement (animals)

The animals used in this study were obtained from the Center of Experimental Animals of Sun Yat-sen University. The animal experimental procedures were performed in accordance with the ARVO Statement for the Use of Animals in Ophthalmic and Vision Research and was approved and monitored by the Institutional Animal Care and Use Committee of Zhongshan Ophthalmic Center (Permit Number: SYXK (YUE) 2014-007).

### Cell culture and tissue sample

(A) Human retinoblastoma cells, the WERI-Rb1 cell line (ATCC, Manassas, VA, USA), the Y79 cells (ATCC, Manassas, VA, USA), human colorectal carcinoma cells and the RKO cell line (ATCC, Manassas, VA, USA), were cultured in Dulbecco's modified Eagle's medium (DMEM, Gibco, CA, USA) supplemented with 10% fetal bovine serum (FBS; Gibco, CA, USA) and 1% penicillin/streptomycin (Gibco, CA, USA) in a humidified 5% CO_2_ incubator. The cells used for the assays were in the exponential growth phase. Trichostatin A (TSA) was obtained from Sigma-Aldrich Corp (Saint Louise, Missouri). hTSP-1 was purchased from R&D Systems (Minneapolis, MN, USA). (B) Primary mice retinal neurocytes were cultured as described previously [33]. It has been provided by the animal center of Zhongshan Ophthalmic Center, Sun Yat-sen University, China (C) Co-culture system cells were cultured in a transwell system (0.4-μM pore size; BD Bioscience, Bedford, MA, USA) in the presence or absence of 25 nM hTSP-1 for 4 days, respectively.

Fresh tissue samples (retinoblastoma) from patients, after diagnosis and verification by oncologists, were obtained from Zhongshan Ophthalmic Center, Sun Yat-sen University. All the patients included in this study required enucleation treatment because of the massive retinoblastoma classified as Group E or some eyes with advanced Group D according to the IIRC (the International Intraocular Retinoblastoma Classification). The samples were collected after a signed consent form was obtained from the patients or their parents/guardians. The present study was approved and monitored by the Institutional Animal Care and Use Committee of Zhongshan Ophthalmic Center, and it adhered to the provisions of the Declaration of Helsinki for research involving human subjects.

### Cell viability assayed by CCK-8

The viability of WERI-Rb1 and Y79 cells and mice retinal neurocytes was assessed by a Cell counting Kit-8 (CCK8) assay (Dojindo, Japan). The CCK8 reagent was added to each well and cells were incubated for 2 h at 37°C. The absorbance (optical density) at 450 nm was measured. Cell viability was determined by the optical density ratio of a treated culture over an untreated control and represented by quantile normalization in log2 scale.

### Real-time RT-PCR

The total RNA of cells or tissues was isolated using TRIzol Reagent (Invitrogen, Carlsbad, CA). Reverse transcription-polymerase chain reaction (RT-PCR) assays were performed according to the manufacturer's protocol for the SYBR Prime Script TM RT-PCR Kit (Takara, China). Real-time PCR was employed to measure the expression of TSP-1 using the Roche 480 system (Roche, USA). Relative target gene expression was quantitated according to the comparative ΔCT method, i.e., normalized to an endogenous control gene, β-actin, and relative to a calibrator after calculating the efficiency coefficient: relative expression = 2-ΔCT, where ΔCT = CT (target gene)-CT (β-actin). The results are presented as the inverse of the normalized Ct value (InvCt) or as the relative fold change compared with an unstimulated control. The following primer pairs were used: for TSP-1, 5′- AAGAGCATCACCCTGTTTGTG -3′ (sense) and 5′- TCTTCTGGTGTGGTTCCAAAG -3′ (antisense); for HDAC4, 5′-GGTTTGAGAGCAGGCAGAAC-3′ (sense) and 5′- CAGAGAATGAGGCCAAGGAG -3′ (antisense); for HDAC8, 5′- CAATGATGCTGTCCTGGGAAT -3′ (sense) and 5′- GGAGAATTTGTGCAGGGACAC -3′ (antisense); for β-actin, 5′-CACCACACCTTCTACAATGAG-3′ (sense) and 5′- GGAGAATTTGTGCAGGGACAC -3′ (antisense).

### Western blot analysis

Cells or tissues were lysed with radio-immunoprecipitation assay buffer. Western blotting was carried out by standard protocols. The following primary antibodies were used: mouse anti-TSP-1 (Abcam, Cambridge, MA); rabbit anti-HDAC4 (Signalway Antibody, Pearland, TX); rabbit anti-GAPDH, rabbit anti-P21, rabbit anti-HDAC8 (Proteintech Group, Chicago, IL); rabbit anti-acetylated histone H3, rabbit anti-acetylated histone H3 (Lys14, 27, 56 and K18), rabbit anti-phospho-H2AX ser-139 (Cell Signaling Technology, Danvers, MA), synaptophysin (Abcam, Cambridge, MA, USA). Proteins were visualized with horseradish peroxidase (HRP)-conjugated anti-rabbit, anti-mouse IgG, (Cell Signaling Technology, Danvers, MA) followed by use of the ECL chemiluminescence system. Western blot data was subjected to densitometry analysis by computerized image analysis and software (Gel-Pro Analyzer software ver.6.0, Media Cyberetics, USA).

### Plasmid construction

The NHEJ reporter plasmid pEPI-NHEJ, used as a substrate for the quantitative NHEJ assay, was derived from pEPI-EGFP (generously provided by Dr. H.J. Lipps), which contains a human scaffold/matrix-attached region (S/MAR) and allows for sustained episomal replication without chromosomal integration in human cells [35]. A 52-bp fragment containing an ISceI recognition site and chromosomally integrated GFP-based reporter was inserted into the unique NheI site of plasmid pEPI-EGFP. The HR reporter plasmid pEPI-HR, used as a substrate for the quantitative HR assay, was derived from pEPI-EGFP and pDRGFP [30] (purchased from Addgene, USA). The S/MAR fragment was obtained from pEPI-EGFP by restriction digestion with BglII and EcorI. The HR fragment was obtained by restriction digestion with SppI and AgeI. Then two fragments were ligated with T4 Ligase. The mitotic stability of the episomal plasmid was determined by quantifying the copy number of the episomal plasmid using real time PCR analysis of extrachromosomal plasmid extracted up to 35 days after transfection.

The plasmid pEPI-GFP-Rb1 was derived from pEPI-GFP [35], which human Rb1 cDNA was inserted in pEPI-GFP by restrictional digested sites, KpnI and BamHI.

### TSP-1 promoter-reporter assay

In each transfection, 2×10^6^ WERI-Rb1 cellsor Y79 cells were electroporated with 10 μg of −2033/+750 human TSP-1 promoter CAT (Addgene, USA). The cells were harvested and assayed after 24 h. At twenty-four hours after transfected cells were maintained in complete RPMI medium, CAT assays (Roche, Indianapolis, IN, USA) were performed according to the manufacturer's instructions.

### Immunohistofluorescence assay

WERI-Rb1 cells, Y79 cells or tissue sections were fixed in methanol and then were characterized by staining with mouse anti-TSP-1 (Abcam, Cambridge, MA, USA) [54], synaptophysin (Abcam, Cambridge, MA, USA and rabbit anti-phospho-H2AX ser-139 (Cell Signaling Technology, Danvers, MA), respectively. Secondary anti-mouse antibodies (CST, Lenexa, KS, USA) were added at room temperature, and the nuclei were stained with DAPI. The cells were counterstained with the fluorescent nuclear-binding label 4,6-diamidino-2-phenylindole. Images were obtained by fluorescence microscopy.

### Quantitative NHEJ and HR assay

Seven days after transfection of pEPI-NHEJ or pEPI-HR into WERI-Rb1 cells or Y79 cells by electroporation, the cells were treated with hTSP-1 and scrambled controls. At twenty-four hours after treatment, pRFP–ISceI–GR (purchased from Addgene, USA) was transfected into the cells. To allow for ISceI expression and NHEJ or HR, the cells were grown in full medium. If NHEJ or HR occurred, the normal expression of GFP could be observed. The intact pEGFP-N1 was used as the positive control, and treatment with PBS and no plasmid was used as the negative control. Forty-eight hours later, the cells were harvested and subjected to two-color fluorescence analysis. The green fluorescent cells represented the efficiency of NHEJ and HR. For each analysis, 200,000 cells were processed.

### Cell cycle assay

WERI-Rb1 cells or Y79 cells were harvested, fixed with 75% ice-cold ethanol in PBS and kept at 4°C. Prior to analysis, cells were washed twice with PBS and then incubated for 30 min in a propidium iodide staining solution containing 0.05 mg/ml propidium iodide, 1 mM ethylene-diaminetetraacetic acid (EDTA), 0.1% Triton X-100™ and 1 mg/ml ribonuclease A (RNase A) (all from Sigma-Aldrich, St. Louis, Missouri). The staining fluorescence intensity was measured using a cytomics FC500 MCL flow cytometer (Beckman Coulter Inc, USA) and used to determine the G1/M ratio.

### Assessment of apoptosis

WERI-Rb1 cells or Y79 cells were harvested and then stained with Annexin V and PI using fluorescein isothiocyanate (FITC)-labeled Annexin V and PI (Annexin-V-PI Kit, Roche, Germany) according to the manufacturer's protocols. Apoptosis was quantified by flow cytometry. A minimum of 10,000 events were collected and analyzed using a FACS Calibur instrument and CellQuest Pro software (Becton Dickinson, USA).

### Murine xenograft model of retinoblastoma

The experimental procedures were approved through the Ethical Committee of Sun Yat-sen University (2014007). Swiss background nu/nu mice were obtained from the Center of Experimental Animals of Sun Yat-sen University and were kept in a conventional environment in cages with filter tops. The WERI-Rb1 cells were re-suspended as a single-cell suspension in PBS. Using a Hamilton needle, 2×10^5^ WERI-Rb1 cells were injected into the vitreous space of the right eyes of nude mice under sterile conditions using a dissecting microscope; the left eyes were used as untreated controls. The mice were observed weekly for tumor development. Two weeks after cell implantation, the nude mice were randomized (6 mice for each group) to a treatment group receiving vitreous injections two times per week of 2 μl of hTSP-1 at a dose of 2 μM or to a control groups receiving balanced salt solution. All of the animals underwent serial ophthalmologic examinations. At day 30 after Weri-Rb1 cell implantation, the globes were enucleated rand fixed in 4% paraformaldehyde for 12 h. They were then were processed into paraffin-embedded sections, and sequential meridian sections (5 μm thick) were creating through the optic disc. The sections were stained with H&E (hematoxylin and eosin).

### Statistical analysis

The data shown are representative of three independent experiments with each experiment performed in triplicate. The data are expressed as the means ± SDs. Statistical analyses were performed using the SPSS for Windows software package, version 10.5 (Chicago, IL, USA). The differences between mean values were evaluated using Student's two-tailed t-test (for two groups) or analysis of variance (ANOVA, for more than two groups). A P-value <0.05 was considered to indicate a statistically significant difference.
